# Novel Application of Ion Mobility Mass Spectrometry Reveals Complex Ganglioside Landscape in Diffuse Astrocytoma Peritumoral Regions

**DOI:** 10.3390/ijms26178433

**Published:** 2025-08-29

**Authors:** Raluca Ica, Mirela Sarbu, Roxana Biricioiu, Dragana Fabris, Željka Vukelić, Alina D. Zamfir

**Affiliations:** 1 National Institute for Research and Development in Electrochemistry and Condensed Matter, 300224 Timisoara, Romaniamaria.biricioiu99@e-uvt.ro (R.B.); 2 Faculty of Physics, West University of Timisoara, 300223 Timisoara, Romania; 3 Department of Chemistry and Biochemistry, School of Medicine, University of Zagreb, 10000 Zagreb, Croatiazvukelic@mef.hr (Ž.V.); 4 Department of Technical and Natural Sciences, Aurel Vlaicu University of Arad, 310130 Arad, Romania

**Keywords:** astrocytoma, peritumoral tissue, gangliosides, ion mobility mass spectrometry, biomarker

## Abstract

Diffuse astrocytoma is a primary brain tumor known for its gradual and diffuse infiltration into the surrounding brain tissue. Given this characteristic, the investigation of the peritumoral region holds potential biological and clinical relevance. In this study, ion mobility spectrometry mass spectrometry (IMS MS) was optimized and applied for the first time for the analysis of gangliosides present in the peritumoral tissue of diffuse astrocytoma. Ganglioside profiling and structural characterization were conducted using high-resolution nanoelectrospray ionization (nanoESI) IMS MS, along with tandem mass spectrometry (MS/MS) via low-energy collision-induced dissociation (CID) in the negative ion mode. Using IMS MS-based separation and screening, we observed a greater diversity of ganglioside species in the peritumoral tissue than previously reported. Notably, an elevated expression was detected for several species, including GT1(d18:1/18:0), GT1(d18:1/20:0), GM2(d18:1/16:2), GD1(d18:1/16:0), GD2(d18:1/20:0), Fuc-GT3(d18:1/24:4), and Fuc-GD1(d18:1/18:2). Although preliminary, these observations prompt consideration of whether these species could be implicated in processes such as microenvironmental modulation, tumor cell infiltration and invasion, maintenance of cellular interactions, or regulation of immune responses. Additionally, their potential utility as biomarkers may merit further exploration. In the subsequent phase of the study, structural analysis using IMS MS, CID tandem MS, and fragmentation data supported the identification of GT1b(d18:1/20:0) isomer in the peritumoral tissue. However, given the exploratory nature of the study and reliance on limited sampling, further investigation across broader sample sets is necessary to extend these findings.

## 1. Introduction

Diffuse astrocytoma is an invasive primary brain tumor, grade 1, according to the World Health Organization (WHO) classification from 2021, characterized by the infiltration of tumor cells with glial differentiation into the adjacent brain areas. Although diffuse astrocytoma has a slower pace of growth than astrocytoma IDH-mutant (grade 2,3,4, WHO 2021) and glioblastoma (grade 4, WHO 2021), in some cases the tumor cell spread in nearby areas is so insidious that there is no clear separation from the normal surrounding brain tissue. This tumor type, predominantly localized in the cerebral hemispheres, affects patients of all ages, being, however, more common in older adults [1]. Depending on the location and size of the tumor, diffuse astrocytoma may cause psychiatric symptoms such as personality changes, cognitive and behavioral disturbances, confusion and hallucinations. For example, dementia associated with diffuse astrocytoma is often preceded by psychiatric symptoms such as confusion and visual hallucinations [2]. Moreover, some studies reported an inverse relationship between Alzheimer’s disease (AD) and this type of cancer, suggesting that individuals with a history of cancer may have a reduced risk of developing AD, and vice versa [3]. This inverse association has been noted in various studies, indicating a complex interplay between the pathophysiological mechanisms of cancer and neurodegenerative diseases [4,5].

Additionally, diffuse astrocytoma leads to cranial nerve paralysis, headaches, cerebral disorders, psychological changes, and motor weakness as the most frequently observed warning signs. Similarly to Parkinson’s disease [6], heart diseases [7,8], and other forms of cancer [9] the depressive disorder, which represents a risk factor for an unfavorable outcome, may also occur in association with astrocytoma [10]. It is important to note that most of the symptoms vary depending on the affected brain region. For instance, in certain brain areas such as the right frontal lobe, large tumors can develop before patients or those around them notice evident symptoms. In other regions, such as the left temporal lobe or left frontal lobe, even a small lesion can lead to a noticeable symptomatology [1].

The diagnostic journey often begins with a thorough neurological examination. Clinicians assess the patient’s cognitive functions, motor skills, sensory perceptions, and reflexes to identify any abnormalities that might suggest the presence of a brain tumor. Symptoms such as headaches, seizures, or changes in personality can prompt further investigation.

Magnetic Resonance Imaging (MRI) stands as the cornerstone of neuroimaging for diffuse astrocytomas. MRI provides high-resolution images of the brain anatomy, allowing for the detailed visualization of tumor size, location, and its relationship with adjacent structures. Advanced MRI techniques enhance the detection of these infiltrative tumors [11,12]. In certain scenarios, Computed Tomography (CT) scans may be employed, especially when MRI is contraindicated. CT imaging is able to reveal mass effects or calcifications associated with the tumor, although it offers less detailed soft tissue contrast in comparison to MRI.

Definitive diagnosis requires histopathological confirmation through a biopsy. Stereotactic needle biopsy or surgical resection allows for the extraction of tumor tissue, which is then examined microscopically to determine the tumor grade and specific histological features. This analysis is crucial for distinguishing diffuse astrocytoma from other gliomas or potential brain lesions [13,14,15].

In recent years, molecular profiling has become increasingly important in the diagnostic process. Identifying genetic mutations, such as *IDH1* or *IDH2* mutations, provides valuable prognostic information and may influence therapeutic decisions. For instance, the presence of IDH mutation is often associated with a better prognosis and may guide the selection of targeted therapies [16]. Consequently, diagnosing diffuse astrocytoma involves a multidisciplinary approach that combines clinical assessments, advanced imaging modalities, histopathological examination, and molecular analyses [17]. Certainly, an early diagnosis of diffuse astrocytoma is crucial as it enables timely intervention that can slow disease progression and improve patient outcomes. Delays in diagnosis may hinder access to treatment, potentially impacting life quality and survival [18].

Considering the characteristics of diffuse astrocytoma, of major importance is the analysis of the peritumoral brain zone, represented by the area surrounding the tumor. This region often harbors infiltrative tumor cells that contribute to recurrence. Exploring the peritumoral zone is vital for determining the extent of resection to prevent recurrence and understand the mechanisms of tumor growth [19]. Hence, a comprehensive examination of both the tumor and its surrounding tissue is of utmost importance for accurate diagnosis and effective treatment planning.

Gangliosides, complex glycosphingolipids abundant in the central nervous system, have emerged as significant biomarkers in the molecular diagnostic landscape of brain tumors [20,21,22,23]. Their unique composition and expression patterns are able to provide valuable information related not only to the tumor presence, but also its type and progression [20]. The specific composition and relative proportion of tumor-associated gangliosides may confer functional advantages that contribute to tumor aggressiveness, and may represent valuable targets for therapeutic intervention or diagnostic development [24].

High-resolution mass spectrometry (HR MS) has proven to be an indispensable tool in this context, offering exceptional sensitivity and specificity for the detailed analysis of ganglioside profiles [25].

In a preliminary study, our group employed HR MS to comparatively map the gangliosides expressed in low-grade astrocytoma vs. its surrounding tissue, and normal brain tissue. The analysis identified a specific pattern associated with each of the three types of tissues. Notably, the ganglioside components discovered in the surrounding tissue exhibited elevated levels of sialylation, fucosylation, and acetylation, which may correlate with tumor expansion into adjacent brain areas [26]. Hence, the integration of HR MS in ganglioside analysis was shown to represent a promising avenue for advancing molecular diagnostics in brain tumors, facilitating early detection and personalized treatment strategies.

The potentials of MS for the discovery of biologically relevant molecules increased in the last decade due to the introduction of ion mobility separation (IMS) coupled to MS (IMS MS). Based on the properties of the transport driven by the electric field, IMS MS is able to separate isomers, isobars and conformers as well as to discriminate minor components in highly complex biological mixtures. Our earlier studies, targeting the first implementation of IMS MS in brain ganglioside research [27,28], indicated that, in conjunction with collision-induced dissociation (CID), IMS MS results in a method of unsurpassed efficiency in detection and structural elucidation of single components in native mixtures and of minor species with diagnostic value.

In this context, we have extended our previous investigations to include the analysis of gangliosides expressed in the peritumoral tissue using the high-performance HR MS platform combining IMS MS and CID tandem MS (MS/MS). This advanced approach allowed for (a) discovery of novel structures due to the efficient separation of ions according to their mobility; and (b) a more detailed characterization of the chemical composition of the tissue surrounding the tumor. Hence, the findings of this study enhanced our knowledge of ganglioside expression in the tissue surrounding astrocytoma, potentially aiding the development of more effective and personalized ganglioside-based therapies.

## 2. Results and Discussions

The nanoelectrospray(nanoESI) IMS MS screening of native ganglioside mixture extracted from peritumoral tissue of diffuse astrocytoma disclosed a diverse range of gangliosides, shedding light on the molecular landscape surrounding this type of tumor. Gangliosides, as an important class of sialic acid-containing glycosphingolipids, are known to play crucial roles in cellular processes such as adhesion, signaling, and immune modulation, all of which being significant in the context of tumor development and invasion in the nearby brain tissue.

**Figure 1:**
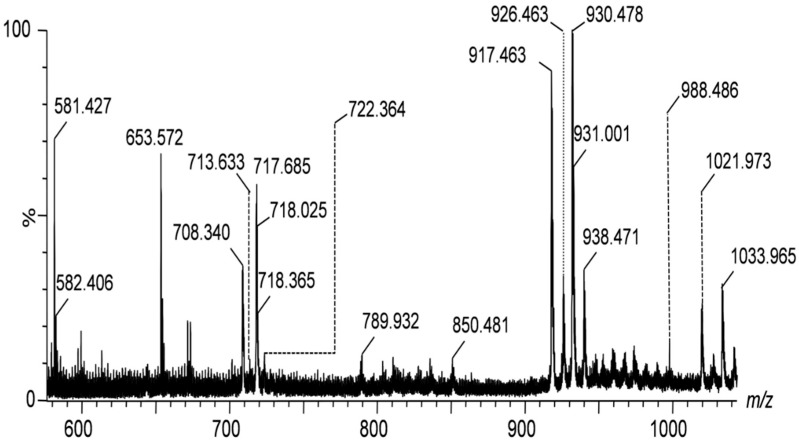
(−)nanoESI IMS MS screening of ganglioside mixture extracted from the peritumoral tissue of diffuse astrocytoma; *m*/*z* range (600–1000). Solvent: methanol; sample concentration: 5 pmol∙μL^−1^; flow rate: 10 μL∙min^−1^; spray voltage: 1.4 kV; cone voltage: 40 V; source block temperature: 100 °C; desolvation temperature: 150 °C. Ion assignment in Table 1.

**Figure 2:**
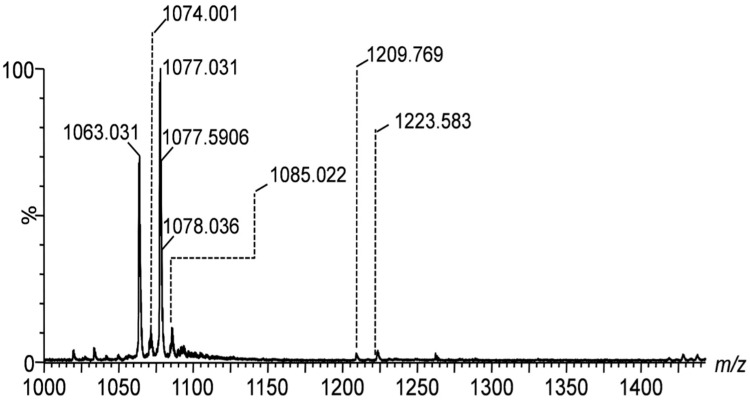
(−)nanoESI IMS MS screening of ganglioside mixture extracted from the peritumoral tissue of diffuse astrocytoma; *m*/*z* range (1000–1400). Conditions as described in Figure 1. Ion assignment in Table 1.

**Figure 3:**
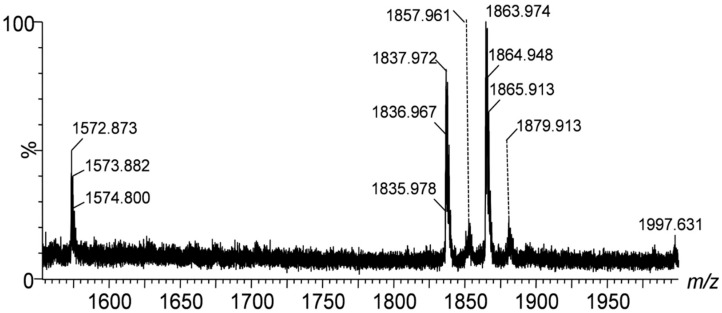
(−)nanoESI IMS MS screening of ganglioside mixture extracted from the peritumoral tissue of diffuse astrocytoma; *m*/*z* range (1600–2000). Conditions as described in Figure 1. Ion assignment in Table 1.

The IMS mass spectra shown in Figure 1, Figure 2 and Figure 3 display the mass-to-charge ratio (*m*/*z*) profiles of gangliosides detected in the peritumoral tissue of the investigated diffuse astrocytoma.

Figure 1 presents the IMS mass spectra of the native ganglioside mixture extracted from the peritumoral tissue of diffuse astrocytoma in the *m*/*z* range of (600–1000), showcasing the distribution of lower molecular weight species, whereas Figure 2 and Figure 3 display the *m*/*z* ranges (1000–1400) and (1600–2000) corresponding to higher molecular weight species.

**Figure 4:**
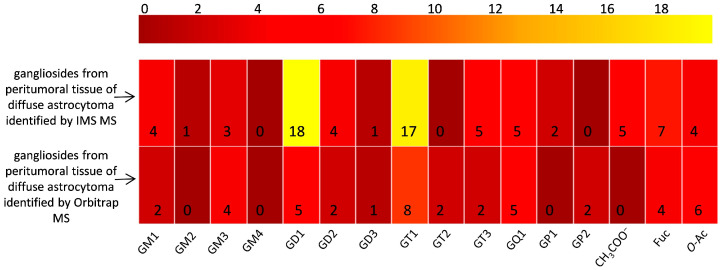
Heatmap illustrating differential detection of ganglioside species in peritumoral tissue of diffuse astrocytoma by IMS MS and Orbitrap MS [26].

The spectra are to be directly correlated with Table 1, which lists the identified gangliosides, their experimental (*m*/*z_exp_*) and theoretical (*m*/*z_theor_*)*m*/*z* values, and the proposed molecular structures. As visible in Table 1, IMS MS revealed in the first place that ganglioside mixture from the peritumoral tissue of diffuse astrocytoma presents various ionization patterns; the components, were detected in multicharged anionic forms such as [M-2H]^2−^ and [M-3H]^3−^, which facilitated their identification and characterization. Secondly, IMS MS data indicated that the gangliosidomeassociated with astrocytoma cell infiltration in the surrounding tissue exhibits a much higher structural complexity than previously known [26]; no less than 64 molecular ions corresponding to 55 distinct ganglioside species were detected in the ganglioside sample from peritumoral tissue. Figure 4 illustrates a comparative heatmap of ganglioside species detected in the peritumoral tissue of diffuse astrocytoma, integrating the findings from IMS MS analysis conducted in the present study with those obtained via Orbitrap MS in our prior investigation [26].

Compared to the previous study using Orbitrap technology, which identified only 40 distinct ganglioside species in tissue adjacent to diffuse astrocytoma, the current IMS MS approach shows an improvement in detection capabilities. The observed increase in the number of identified species reveals an enhanced sensitivity and a broader analytical range, due to the high separation efficiency of IMS MS. This improvement enables a more detailed profiling of the gangliosidome in peritumoral tissue. The ability to detect a wider variety of gangliosides may be particularly relevant in the context of astrocytoma cell infiltration. Overall, the data indicate that IMS MS can refine ganglioside characterization and may support a deeper understanding of their potential role in tumor progression.

By IMS MS the monosialylated(GM), disialylated(GD), trisialylated(GT), and tetrasialylated(GQ)glycoforms and particularly GM1(d18:1/18:1) and GM2(d18:1/16:2) were found prominently expressed in the tissue surrounding the tumor. The latter species are often associated with cellular adhesion and immune evasion mechanisms [29], which are crucial for the survival and spreading of tumor cells. Disialylated gangliosides such as GD1(d18:1/16:0) and GD2(d18:1/20:0), known to have been implicated in processes enhancing tumor invasion and resistance to apoptosis [30,31], were also detected through MS signals of relatively high abundance.

Figure 2, covering the range of *m*/*z* (1000–1400), captures higher mass gangliosides such as the tri- and tetrasialotetraoses GT1 and GQ1. The signals at *m*/*z* 1033.965, 1077.031, and 1223.583 correspond to the species GT1(d18:1/14:1), GT1(d18:1/20:0), and GQ1(d18:0/20:0). This identification is supported by the high mass accuracy, i.e., only 9.29 ppm mass deviation for GT1(d18:1/20:0), which indicates the precision and robustness of the detection method.

Figure 3, depicting the highest molecular weight gangliosides, features key signals at *m*/*z* 1835.978, 1863.974, and 1879.913, which correspond to GD1(d18:1/18:0), GD1(d18:1/20:0), and GD1(d18:0/19:0), respectively. The highly accurate mass measurements, with deviations as low as 7.63 ppm for GD1(d18:1/18:0), demonstrate again the consistency of the IMS MS measurements and ganglioside assignment.

The class of trisialylated gangliosides, including GT1(d18:1/18:0) and GT1(d18:1/20:0), was observed in notable abundance. These ganglioside species are essential to neuronal interactions being likely to influence the microenvironment surrounding the tumor. The detection of tetrasialylated gangliosides, such as GQ1(d18:1/16:3), emphasizes the complexity of the glycosylation patterns in the peritumoral region. Additionally, fucosylated ganglioside variants such as Fuc-GT3(d18:1/24:4) and Fuc-GD1(d18:1/18:2) were discovered; these species are often linked to an enhanced tumorigenic potential and immune suppression [32,33].

The high-resolution nature of IMS MS provided accurate mass measurements with minimal deviations, supporting the reliability of signal assignment to ganglioside components. Hence, the average mass accuracy of 11.38 ppm, offers a high confidence in molecular identification.

**Figure 5:**
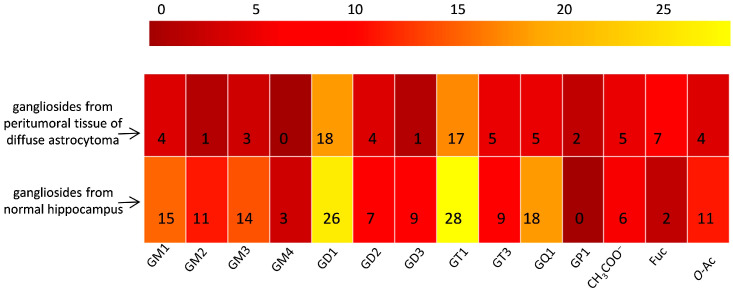
IMS MS-derived heatmap depicting distinct ganglioside expression profiles in peritumoral tissue of diffuse astrocytomavs. normal hippocampus used as a control.

A comparative IMS MS analysis performed under similar conditions on histologically normal adult human hippocampal tissue [34] selected here as control due to its well-characterized lipid and ganglioside profiles by IMS MS and its availability from non-oncological contexts, revealed a notably different gangliosidic profile, both in terms of composition and molecular diversity. In total, 140 ganglioside species were detected in the healthy tissue, nearly triple the number identified in the peritumoral sample. Figure 5 presents a heatmap delineating the distinct expression profiles of ganglioside species in peritumoral tissue of diffuse astrocytoma compared to the normal hippocampal tissue serving as a control.

Obviously, structural features specific to the normal hippocampal gangliosides included a higher proportion of species with shorter fatty acid chains and increased presence of trihydroxylatedsphingoid bases, suggesting a specific ceramide architecture. Furthermore, the healthy tissue exhibited improved separation across charge states, *m*/*z* values, glycan chain lengths, and degrees of sialylation, with reduced spectral overlap. These observations suggest a substantial gangliosidic remodeling occurring in the peritumoral microenvironment and confirm the capability of IMS MS to resolve and distinguish pathophysiological alterations in ganglioside expression patterns at a high level of structural resolution.

From a biological standpoint, these gangliosides may play a role in the peritumoral region, although their specific functions remain to be fully elucidated. The monosialylated forms could potentially interact with components of the extracellular matrix, thereby influencing processes such as tumor cell adhesion and migration [29]. Disialylated and trisialylated gangliosides might also participate in signaling pathways that affect proliferation and invasion [35], though this aspect remains speculative. Fucosylated and highly sialylated species are of particular interest due to their reported ability to modulate immune responses, possibly by dampening the activity of immune effector cells and contributing to tumor immune evasion [36,37].

In the case of diffuse astrocytoma, the peritumoral tissue represents a key interface between the tumor and adjacent normal brain tissue, making it a highly relevant area for investigation. The presence of various ganglioside species in this region may point to their involvement in processes that support tumor expansion and interaction with surrounding tissue. For instance, they may contribute to modifying the physical characteristics of the extracellular matrix, potentially influencing tumor cell migration and invasion.

It is important to acknowledge the limitations of the current study, particularly the challenges associated with obtaining peritumoral tissue and the reliance on a single biopsy sample. Both the measurements and the biological interpretations presented here are preliminary and should be viewed with caution. Further validation through IMS MS studies involving additional samples will be essential to confirm these initial observations and clarify the functional roles of these gangliosides.

To determine the detailed structure of the dominant species and the position isomers related to Neu5Ac, we have chosen the doubly charged precursor ion [M-2H^+^]^2−^ detected at *m*/*z* 1077.031 as an example to illustrate the fragmentation analyses carried out by CID MS/MS. According to the exact mass calculation (*m*/*z_theor_* 1077.041), this ion was assigned with a mass accuracy of 9.29 ppm to the trisialotetraose GT1(d18:1/20:0) species.

For the CID MS/MS structural investigation, the precursor ion was isolated within a 1 *m*/*z* window and subjected to fragmentation using a broad range of collision energies ramped from 20 and 45 eV to facilitate the production of diagnostic fragment ions through glycosidic bond and cross-ring cleavages. All tandem mass spectra were generated by summing up the scans acquired over 2 min across the entire range of applied collision energies.

**Figure 6:**
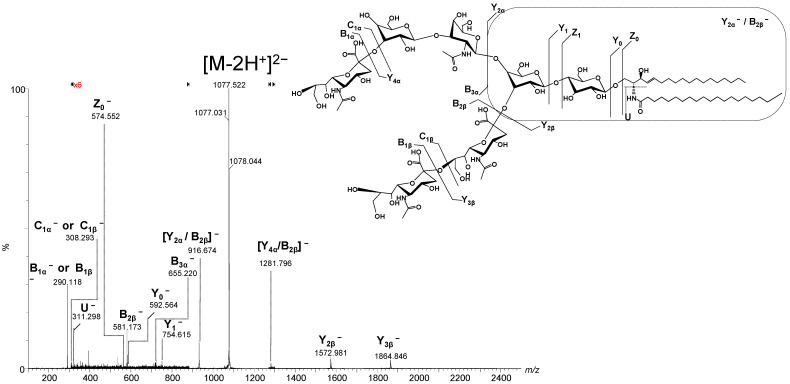
(−)nanoESI IMS CID MS/MS of the doubly charged [M-2H^+^]^2^− ion detected at *m*/*z* 1077.031, corresponding to the GT1(d18:1/20:0) ganglioside species isolated and fragmented from ganglioside mixture in peritumoral tissue of diffuse astrocytoma. CID at variable collision energy within the range of 20–45 eV. Inset: fragmentation scheme of GT1(d18:1/20:0) under the employed CID conditions. The fragmentation pattern reveals structural details of the species, highlighting the position of Neu5Ac residues.

Figure 5 presents the fragmentation mass spectrum of the doubly charged precursor ion [M-2H^+^]^2−^ detected at *m*/*z* 1077.031, while Figure 6 illustrates the dissociation scheme experienced by this ion under the employed sequencing conditions.

Based on the detailed fragmentation analysis presented in Figure 5, the generated sequence ions document the saccharide chain structure, sialylation status, the positions along the chain of the three Neu5Ac monosaccharides, and the overall ceramide composition.

The sialylation status of the molecule is documented by the high-intensity signal at *m*/*z* 290.118 attributed to the cleaved Neu5Ac residue generating B_1α_^−^ and/or B_1β_^−^ ions, along with their less abundant C_1α_^−^ and/or C_1β_^−^ counterparts at *m*/*z* 308.293 and the ion corresponding to the detachment of the disialo group (Neu5Ac)^2−^ detected at *m*/*z* 581.173 as the monocharged B_2β_^−^ ion. Further, the B_3α_^−^ ion at *m*/*z* 655.220 is consistent with the Neu5Ac–Gal–GalNAc trisaccharide motif of the non-reducing end. Subsequently, a series of ions fully characterize the reducing end as well. Hence, the Gal-Glc disaccharide sequence in the proximity of the aglycone, the Gal–Glc–Cer motif at the reducing end, and the (d18:1/20:0) composition of the ceramide are supported by the following ions: Y_1_^−^ at *m*/*z* 754.615, Y_0_^−^ at *m*/*z* 592.564, Z_0_^−^ at *m*/*z* 574.552, and the double cleavage [Y_2α_/B_2β_]^−^ at *m*/*z* 916.674, respectively. In addition, [Y_4α_/B_2β_]^−^ detected at *m*/*z* 1281.796 confirms the Gal–GalNAc–Gal–Glc–Cer oligosaccharide backbone (Figure 5).

Based on the detailed fragmentation analysis, which includes the identification of key diagnostic ions and the confirmation of the Neu5Ac–Gal–GalNAc sequence, we have successfully determined the structure of the GT1b isomer. Due to the inherent molecular symmetry of the oligosaccharide core, it is not possible to completely rule out the presence of the GT1c isomer, as the structural resemblance between the two makes their differentiation particularly challenging. Although the characteristic Neu5Ac–Neu5Ac–Neu5Ac–Glc–Cer fragment of GT1c was not detected, its absence alone does not provide definitive evidence against this isomer. A decisive factor in our structural assignment was the ion mobility mass spectrometry profile, which revealed the presence of a single isomer. The observation of a single drift time, along with the corresponding collision cross-section values, strongly supports the conclusion that the analyzed compound corresponds to GT1b, as no additional isomeric species were detected.

## 3. Materials and Methods

### 3.1. Peritumoral Tissue

A 30-year-old male patient was hospitalized for evaluation after experiencing epileptic seizures characterized by loss of consciousness and involuntary contractions of the right arm. These episodes occurred several times a day, each lasting a few minutes. Apart from these symptoms, the patient was in generally good health. Neuroradiological investigations, including computed tomography and magnetic resonance imaging, revealed an expansive lesion measuring 38 × 49 mm in the left precentral parasagittal frontal region of the brain. The lesion had poorly defined borders and exerted a significant compressive effect on the surrounding tissue. Surgical intervention was performed to remove the tumor along with a portion of adjacent tissue. Histopathological analysis of the excised tumor revealed high cellular density, minimal nuclear pleomorphism, the presence of microcysts (small fluid-filled spaces), and abundant intercellular edema. Based on these findings, the tumor was classified as a diffuse astrocytoma, grade I, according to the WHO 2021 classification of adult brain tumors.

Permission for the use of human tissues for scientific research was obtained from the Ethics Committee of the Zagreb Medical Faculty under the Project “Structure-function glycolipidomics of brain development and malignant alteration”, No.108-1081870-2415, funded by the Croatian Ministry of Science, Education and Sport. The patient provided informed consent for participation in the study.

### 3.2. Extraction and Purification of Gangliosides

A crude ganglioside mixture was extracted and purified from astrocytoma peritumoral tissue using a standardized protocol under consistent experimental conditions.

The extraction method was based on the procedure developed by Svennerholm and Fredman [38], later modified by Vukelić et al. [39], and consistently applied in our previous studies on brain tumor gangliosides [40,41,42]. In brief, the tissue samples were weighed and homogenized in ice-cold distilled water to prepare a 10% homogenate. Lipids were extracted twice using a solvent mixture of chloroform and methanol (1:2, *v*:*v*). The extraction process involved partitioning and repartitioning by adding chloroform, methanol, and water until a final volume ratio of 1:1:0.8 was reached. The combined upper phases, which contained the gangliosides, were collected for further processing. The crude ganglioside extract was subjected to several purification steps: (a) precipitation of co-extracted protein–salt complexes followed by centrifugation; (b) removal of low molecular weight contaminants through gel-filtration using a Sephadex G-25 column; (c) and overnight dialysis against water at 4 °C to eliminate the remaining impurities. The purified extract was subsequently evaporated to complete desiccation in a SpeedVac SPD 111V system (Savant, Düsseldorf, Germany) to yield the final ganglioside mixture.

A stock solution of the ganglioside extract (~1 mg/mL) was prepared by dissolving the dried material in pure methanol and stored at −20 °C. For IMS MS and CID MS/MS analyses, the stock solution was diluted in pure methanol to render the working aliquots at a concentration of approximately 5 pmol∙μL^−1^ (calculated based on an average molecular weight of 2000 g/mol). Prior to ion mobility mass spectrometry analysis, the sample solution was centrifuged for 5 min at 6000 rpm using a mini-centrifuge (Thermo Fisher Scientific, Waltham, MA, USA). The resulting supernatant was collected and subjected to (−)nanoESI IMS MS and MS/MS analysis using CID at low collision energies. All reagents used in this procedure were of analytical grade and sourced from Merck (Darmstadt, Germany).

### 3.3. Ion Mobility Mass Spectrometry

For the ion mobility mass spectrometry experiments conducted on the native gangliosides extracted from the peritumoral tissue of diffuse astrocytoma in negative ionization mode, a Synapt G2-S mass spectrometer (Waters, Manchester, UK) equipped with a nanoESI source was employed. Data acquisition and IMS data processing were performed using Waters MassLynx (version V4.1, SCN 855) and Waters Driftscope (version V2.7) software, both running on a connected PC. A 30 µL sample solution at a concentration of 5 pmol/µL in methanol was infused in the mass spectrometer at a 10 µL∙min^−1^ flow rate, through 10 cm long capillaries with 10 µm tip sizes and 4 mm taper lengths. A steady spray, an efficient ionization and a minimum in-source fragmentation were achieved at 1.4 kV applied to the capillary through a platinum wire and 40 V applied to the cone. The source block temperature was kept at 100 °C, whereas the desolvation temperature at 150 °C and the desolvation gas flow rate at 800 L·h^−1^.

In the IMS MS separation region, the ions were introduced as pulses. The continuous ion beam generated by nanoESI was first accumulated in an ion funnel, then converted into a pulsed beam, and periodically injected into the traveling wave ion guide. The ions then passed through the quadrupole region into the drift region, which was filled with the drift gas, allowing separation based on ion mobility. Finally, the ions entered the time-of-flight (TOF) analyzer, where they were separated according to their mass-to-charge (*m*/*z*) ratios.

The traveling-wave technology, employed in this setup, involved a stacked-ring ion guide with an applied traveling voltage wave. In the drift tube, the ions moved through a nitrogen buffer gas and were propelled forward by the wave. Smaller ions with lower collision cross sections experienced less friction and advanced more steadily compared to larger ions, which fell behind and were caught by subsequent waves. The parameters influencing the separation, such as the ion mobility cell gas pressure, wave amplitude, and wave velocity, were optimized as follows: 90 mL/min IM gas flow, 650 m/s IM wave velocity, and 40 V IM wave height.

The dried native ganglioside mixture dissolved in methanol to the concentration of 5 pmol∙μL^−1^ was loaded in the nanoESI capillary and infused into the IMS MS instrument. The sample was profiled in the negative ion mode nanoESI IMS MS with the signal acquired for 3 min.

CID experiments were performed after mobility separation, with collision energies ramped from 20 to 45 eV to ensure a comprehensive sequence ion coverage. This setup provided a robust separation and a reliable structural elucidation of sample components.

For each MS and MS/MS experiment, the number of replicates was three. The in-run reproducibility was 100% while the day-to-day reproducibility was 99%.

### 3.4. Ganglioside Abbreviation, Nomenclature and Assignment of the Mass Spectra

Gangliosides were abbreviated according to the system of Svennerholm (1980) [43] and the recommendations of IUPAC-IUB Commission on Biochemical Nomenclature (IUPAC-IUB 1998) [44] as follows:

GM1-II^3^-α-Neu5Ac-Gg_4_Cer; GM2-II^3^-α-Neu5Ac-Gg_3_Cer; GM-II^3^-α-Neu5Ac-LacCer; GD1-II^3^-α-(Neu5Ac)_2_-Gg_4_Cer; GD2-II^3^-α-(Neu5Ac)_2_-Gg_3_Cer; GD3-II^3^-α-(Neu5Ac)_2_-LacCer; GT1-II^3^-α-(Neu5Ac)_3_-Gg_4_Cer; GT2-II^3^-α-(Neu5Ac)_3_-Gg_3_Cer; GT3-II^3^-α-(Neu5Ac)_3_-LacCer, GT4-II^3^-α- (Neu5Ac)_3_-GgCer; GQ1-II^3^-α-(Neu5Ac)_4_-Gg_4_Cer.

Spectral signals were assigned to specific ganglioside species through exact mass determination, achieved by comparing the experimentally obtained *m*/*z* values to the theoretical values calculated using monoisotopic masses. This assignment was further supported by previously established structural data on this class of glycoconjugates [25,26,27,28,34] and by reference to known biosynthetic pathways. MS/MS validation experiments were carried out for 59% of the ions (a few examples are presented in Supplementary Figures S1–S5). The fragment ions corresponding to the sequencing of the glycan core by CID were assigned according to the nomenclature introduced by Domon and Costello [45] and revised later by Costello et al. [46]. The fragment ions associated with the cleavages of the ceramide were denoted according to the dedicated the nomenclature proposed by Ann and Adams [47]. The basic statistical data analyses, including the heat map, were performed using Python3.13 software.

## 4. Conclusions

Diffuse astrocytoma often appears relatively benign under the microscope; however, its tendency to infiltrate surrounding brain tissue presents ongoing clinical challenges. An emerging focus in the field is the molecular characterization of peritumoral tissue, which appears histologically normal and may nonetheless exhibit molecular alterations associated with tumor dissemination. Understanding the peritumoral zone is particularly important given the infiltrative nature of astrocytoma. While conventional histopathology provides valuable diagnostic information, it may not always detect infiltrating tumor cells. The advanced molecular techniques, however, offer a complementary approach by identifying certain molecular structures that might suggest subtle tumor presence beyond the main lesion.

Screening the peritumoral tissue on a molecular level may also offer insights into tumor biology and the early stages of transformation. Detecting relevant markers in these regions could support decisions about surgical margins or the consideration of adjuvant therapy, particularly in cases where histology alone is inconclusive. Moreover, investigating molecular heterogeneity of the peritumoral tissue may contribute to understanding how astrocytomasevolve and potentially resist treatment. In this context, the present study employed for the first time one of the most advanced analytical methods available nowadays for molecular screening, i.e.,ion mobility spectrometry, to offer a detailed analysis of the gangliosides associated with astrocytoma peritumoral tissue.

A prominent outcome of this research was the marked enrichment of trisialylated gangliosides, which are usually involved in neuronal interactions, hence they may influence tumor-related processes such as adhesion, migration, and proliferation. Their elevated expression indicates a potential role in modulating the extracellular matrix, and a contribution to a microenvironment supporting tumor growth and invasion. In addition to trisialylated gangliosides, other significant ganglioside classes were identified, including monosialylated(GM), disialylated(GD), and tetrasialylated(GQ) forms. GM1(d18:1/18:1) and GM2(d18:1/16:2) were previously found to be strongly linked to immune evasion and cellular adhesion, while disialylated species such as GD1(d18:1/16:0) and GD2(d18:1/20:0) are known to enhance tumor invasion and apoptosis resistance. The detection of fucosylated variants, including Fuc-GT3(d18:1/24:4) and Fuc-GD1(d18:1/18:2), suggests a potential role in tumor progression, as such molecules are frequently linked to enhanced tumorigenic properties and immunosuppressive effects.

Further structural analysis through a highly efficient fragmentation technique confirmed the identity and composition of key gangliosides. The fragmentation of the doubly charged precursor ion at *m*/*z* 1077.031, exemplified in this analysis, facilitated the unambiguous identification of the trisialylated GT1(d18:1/20:0) species with a high mass accuracy of 9.29 ppm. CID MS/MS provided detailed information on the saccharide chain structure, sialylation pattern, and, most of all, the positioning of the three Neu5Ac monosaccharides leading to isomer identification. The detection of diagnostic ions allowed for the confirmation of the Neu5Ac–Gal–GalNAc sequence and led to the identification of the GT1b isomer.

The current data provide preliminary indications that gangliosides might be involved in shaping the peritumoral microenvironment, potentially through effects on extracellular matrix composition, cell interactions, and immune modulation. While their possible role in diffuse astrocytoma progression is of interest, it remains to be clarified whether they could have relevance as biomarkers or therapeutic targets. Further comprehensive studies, ideally involving patient-matched adjacent tissue where feasible and diverse patient cohorts, are needed to quantify ganglioside expression across astrocytoma grades and to better understand their contribution to tumor biology. Such investigations may, in the long term, offer insights that could direct future therapeutic strategies. Certainly, while still an evolving area of research, by complementing the traditional histopathology, the molecular screening of peritumoral tissues may provide a new direction for diagnostic and therapeutic approaches. Ongoing investigations will further elucidate the clinical applicability and constraints of this concept and methodology in routine practice.

## Figures and Tables

**Table 1:** Assignment of the ionic species in Figure 1, Figure 2 and Figure 3.

No	*m*/*z_exp_*	*m*/*z_theor_*	Mass Accuracy (ppm)	Proposed Structure *	Molecular Ion
1.	674.863	674.870	10.39	GM2(d18:1/16:2)	[M-2H^+^]^2−^
2.	708.340	708.346	8.47	GT1(d18:1/18:0)^#^	[M-3H^+^]^3−^
3.	713.663	713.679	22.44	GT1(t18:1/18:0)	[M-3H^+^]^3−^
		713.6591	5.47	GT1(d18:1/16:0)	[M-5H^+^+2Na]^3−^
4.	717.685	717.692	9.76	GT1(d18:1/20:0)^#^	[M-3H^+^]^3−^
5.	722.364	722.351	18.01	GT1(t18:1/20:1)	[M-3H^+^]^3−^
6.	727.041	727.033	11.01	GT1(d18:1/22:0)^#^	[M-3H^+^]^3−^
7.	735.694	735.682	15.51	(CH3COO-) GT1(d18:1/18:0)^#^	[M-3H^+^+Na^+^]^3−^
8.	770.928	770.920	10.39	GM1(d18:1/18:1)^#^	[M-2H^+^]^2−^
9.	782.906	782.919	16.62	GM1(d18:1/18:0)^#^	[M-3H^+^+Na^+^]^2−^
10.	785.932	785.944	15.29	GM1(d18:1/20:0)^#^	[M-2H^+^]^2−^
11.	789.952	789.965	16.48	GD3(d18:1/26:1)^#^	[M-2H^+^]^2−^
12.	794.012	794.018	7.56	GQ1(d18:1/16:3)^#^	[M-3H^+^]^3−^
13.	822.422	822.434	14.60	GD2(d18:1/16:0)^#^	[M-2H^+^]^2−^
14.	828.444	828.452	9.66	GD2(d18:0/18:0)	[M-2H^+^]^2−^(-H_2_O)
15.	831.397	831.392	6.02	GQ1(d18:1/22:0)^#^	[M-3H^+^]^3−^
16.	850.481	850.465	18.82	GD2(d18:1/20:0)^#^	[M-2H^+^]^2−^
17.	863.462	863.474	13.90	GD2(d18:1/22:1)	[M-2H^+^]^2−^
18.	902.122	902.110	13.30	GP1(d18:1/18:0)^#^	[M-3H^+^]^3−^
19.	903.468	903.460	8.86	GD1(d18:1/16:0)^#^	[M-2H^+^]^2−^
20.	916.460	916.470	10.92	GD1(d18:1/18:1)^#^	[M-2H^+^]^2−^
21.	917.463	917.475	13.09	GD1(d18:1/18:0)^#^	[M-2H^+^]^2−^
22.	926.463	926.483	21.60	GD1(t18:0/18:0)	[M-2H^+^]^2−^
		926.453	10.80	GD1(d18:1/18:2)	[M-3H^+^+Na^+^]^2−^
23.	930.478	930.486	8.60	GD1(d18:1/20:1)^#^	[M-2H^+^]^2−^
24.	938.471	938.483	13.75	GD1(t18:1/20:1)	[M-2H^+^]^2−^
		938.452	19.62	GD1(d18:1/18:1)	[M-4H^+^+2Na^+^]^2−^
25.	950.477	950.4839	7.26	O-Ac-GD1(d18:1/20:2)^#^	[M-2H^+^]^2−^
		950.4708	6.53	O-Ac-GT3(d18:1/22:1)	[M-4H^+^+2Na]^2−^
26.	951.493	951.488	5.26	*O*-Ac-GD1(d18:1/20:1)	[M-2H^+^]^2−^
27.	958.468	958.476	8.35	(CH_3_COO^−^) GD1(d18:1/18:0)	[M^−^-2H^+^+Na^+^]^2−^
28.	965.478	965.484	6.22	(CH_3_COO^−^) GD1(d18:1/19:0)	[M^−^-2H^+^+Na^+^]^2−^
29.	988.486	988.4919	5.97	Fuc-GD1(d18:1/18:2)	[M-2H^+^]^2−^
30.	991.508	991.512	4.04	Fuc-GT3(d18:1/24:4)^#^	[M-2H^+^]^2−^
		991.502	6.05	Fuc-GT3(d18:1/24:3)	[M-2H^+^]^2−^
31.	1003.509	1003.513	3.99	Fuc-GD1(d18:1/20:1)	[M-2H^+^]^2−^
32.	1009.503	1009.512	8.92	(CH_3_COO^−^) Fuc-GT3(d18:1/22:2)^#^	[M^−^-H^+^]^2−^
		1009.498	4.96	(CH_3_COO^−^) GD1(d18:1/24:2)	[M^−^-3H^+^+2Na^+^]^2−^
33.	1013.486	1013.496	9.87	Fuc-GD1(d18:1/20:2)	[M-3H^+^+Na^+^]^2−^
34.	1018.951	1018.963	11.79	GT1(d18:1/12:2)^#^	[M-2H^+^]^2−^
35.	1021.973	1021.987	13.71	GT1(d18:0/12:0)	[M-2H^+^]^2−^
36.	1024.523	1024.536	12.70	Fuc-GD1(d18:1/23:1)^#^	[M-2H^+^]^2−^
37.	1027.953	1027.969	15.58	GT1(t18:1/12:1)^#^	[M-2H^+^]^2−^
38.	1029.972	1029.954	17.49	GT1(d18:1/12:2)	[M-3H^+^+Na^+^]^2−^
39.	1032.963	1032.979	15.50	GT1(d18:1/14:2)^#^	[M-2H^+^]^2−^
40.	1033.965	1033.987	21.30	GT1(d18:1/14:1)^#^	[M-2H^+^]^2−^
41.	1042.984	1042.962	21.11	GT1(d18:1/14:3)	[M-3H^+^+Na^+^]^2−^
42.	1048.996	1049.010	13.35	GT1(d18:1/16:0)	[M-2H^+^]^2−^
43.	1062.004	1062.018	13.18	GT1(d18:1/18:1)^#^	[M-2H^+^]^2−^
44.	1063.031	1063.026	4.42	GT1(d18:1/18:0)^#^	[M-2H^+^]^2−^
45.	1074.009	1074.017	7.45	GT1(d18:1/20:3)^#^	[M-2H^+^]^2−^
		1074.018	8.38	GT1(d18:1/20:0)	[M-2H^+^]^2−^
46.	1077.031	1077.041	9.29	GT1(d18:1/20:0)^#^	[M-2H^+^]^2−^
47.	1081.997	1082.012	13.86	*O*-Ac-GT1(d18:1/18:2)^#^	[M-2H^+^]^2−^
48.	1085.022	1085.008	12.90	GT1(d18:1/18:0)^#^	[M-4H^+^+2Na^+^]^2−^
		1085.039	15.67	GT1(t18:1/20:0)^#^	[M-2H^+^]^2−^
49.	1088.018	1088.030	11.03	GT1(d18:1/20:0)	[M-3H^+^+Na^+^]^2−^
50.	1095.982	1095.997	13.70	GT1(d18:1/20:3)^#^	[M-4H^+^+2Na^+^]^2−^
51.	1099.065	1099.054	10.01	GT1(t18:1/22:0)	[M-2H^+^]^2−^
52.	1139.658	1139.666	7.02	GM3(t18:1/14:0)	[M-H^+^]^−^
53.	1151.724	1151.705	16.51	GM3(d18:1/16:0)	[M-2H^+^]^2−^
54.	1209.769	1209.783	11.58	GM3(d18:0/20:0)^#^	[M-2H^+^]^2−^
55.	1223.583	1223.597	11.45	GQ1(d18:0/20:0)^#^	[M-2H^+^]^2−^
56.	1227.563	1227.577	11.41	GQ1 (t18:1/18:0)	[M-3H^+^+Na^+^]^2−^
57.	1310.635	1310.642	5.42	Fuc-GQ1(d18:0/22:0)	[M-2H^+^]^2−^
58.	1382.140	1382.152	8.68	GP1(d18:0/22:0)	[M-2H^+^]^2−^
59.	1572.873	1572.900	17.18	GM1(d18:1/20:0)^#^	[M-H^+^]^−^
60.	1835.978	1835.964	7.63	GD1(d18:1/18:0)	[M-H^+^]^−^
61.	1857.961	1857.946	8.08	GD1(d18:1/18:0)^#^	[M-2H^+^+Na^+^]^−^
62.	1863.974	1863.995	11.27	GD1(d18:1/20:0)^#^	[M-H^+^]^−^
63.	1873.961	1873.973	6.41	GD1(d18:0/19:0)	[M-2H^+^+Na^+^]^−^
64.	1879.913	1879.923	5.32	GD1(d18:1/18:0)	[M-3H^+^+2Na^+^]

* d-dihydroxylatedsphingoid base; t-trihydroxylatedsphingoidbase. ^#^ ion composition confirmed by CID MS/MS.

## Data Availability

The original contributions presented in this study are included in the article/supplementary material. Further inquiries can be directed to the corresponding author.

## Supplementary Materials

The following supporting information can be downloaded at: https://www.mdpi.com/article/10.3390/ijms26178433/s1.

## References

[1] Lang M., Colby S., Ashby-Padial C., Bapna M., Jaimes C., Rincon S.P., Buch K. (2024). An imaging review of the hippocampus and its common pathologies. J. Neuroimaging.

[2] Madhusoodanan S., Ting M.B., Farah T., Ugur U. (2015). Psychiatric aspects of brain tumors: A review. World J. Psychiatry.

[3] Realmuto S., Cinturino A., Arnao V., Mazzola M.A., Cupidi C., Aridon P., Ragonese P., Savettieri G., D’Amelio M. (2012). Tumor diagnosis preceding Alzheimer’s disease onset: Is there a link between cancer and Alzheimer’s disease?. J. Alzheimers Dis..

[4] Lanni C., Masi M., Racchi M., Govoni S. (2021). Cancer and Alzheimer’s disease inverse relationship: An age-associated diverging derailment of shared pathways. Mol. Psychiatry.

[5] Xia S., Chen H., Tang T. (2023). Risk of death from Alzheimer’s disease associated with brain tumor, glioma, and glioblastoma. J. Alzheimers Dis..

[6] Ahmad M.H., Rizvi M.A., Ali M., Mondal A.C. (2023). Neurobiology of depression in Parkinson’s disease: Insights into epidemiology, molecular mechanisms and treatment strategies. Ageing Res. Rev..

[7] Bondar L.I., Osser B., Miuța C.C., Petran D., Baltean A.I., Butari D.B., Mariș M.A., Piroș L.E., Almășan R., Gavrila-Ardelean M. (2024). Gender-specific insights into depression in patients with ischemic heart disease: Findings from a pilot study using a self-developed questionnaire. Diseases.

[8] Bondar L.I., Iovanovici D.C., Măduța V., Butari D.B., Șandor F.M., Mariș M.A., Piroș L.E., Miuța C.C., Toderescu C.D., Popescu M.I. (2025). Screening depression in ischemic heart disease: Gender differences and psychosocial implications using a self-developed questionnaire. J. Clin. Med..

[9] Battat M., Omair N., WildAli M.A., Alkaissi A., Amer R., Koni A.A., Salameh H.T., Zyoud S.E. (2024). Assessment of depression symptoms among cancer patients: A cross-sectional study from a developing country. Sci. Rep..

[10] Starkweather A.R., Sherwood P., Lyon D.E., McCain N.L., Bovbjerg D.H., Broaddus W.C. (2011). A biobehavioral perspective on depressive symptoms in patients with cerebral astrocytoma. J. Neurosci. Nurs..

[11] Alshoabi S.A., Alareqi A.A., Omer A.M., Suliman A.G., Daqqaq T.S. (2020). Diffuse astrocytoma and the diagnostic dilemma of an unusual phenotype: A case report. Radiol. Case Rep..

[12] Kim M.M., Parolia A., Dunphy M.P., Venneti S. (2016). Non-invasive metabolic imaging of brain tumours in the era of precision medicine. Nat. Rev. Clin. Oncol..

[13] Tonn J.C., Teske N., Karschnia P. (2024). Astrocytomas of the spinal cord. Neuro-Oncol. Adv..

[14] Khandwala K., Mubarak F., Minhas K. (2021). The many faces of glioblastoma: Pictorial review of atypical imaging features. Neuro-Radiol. J..

[15] Weller M., van den Bent M., Hopkins K., Tonn J.C., Stupp R., Falini A., Cohen-Jonathan-Moyal E., Frappaz D., Henriksson R., Balana C. (2014). EANO guideline for the diagnosis and treatment of anaplastic gliomas and glioblastoma. Lancet Oncol..

[16] Camelo-Piragua S., Jansen M., Ganguly A., Kim J.C., Louis D.N., Nutt C.L. (2010). Mutant *IDH1*-specific immunohistochemistry distinguishes diffuse astrocytoma from astrocytosis. Acta Neuropathol..

[17] Kapoor M., Gupta V. (2025). Astrocytoma. StatPearls.

[18] Brennan P.M., Butler H.J., Christie L., Hegarty M.G., Jenkinson M.D., Keerie C., Norrie J., O’Brien R., Palmer D.S., Smith B.R. (2021). Early diagnosis of brain tumours using a novel spectroscopic liquid biopsy. Brain Commun..

[19] Grishin A.S., Achkasova K.A., Kukhnina L.S., Sharova V.A., Ostapyuk M.V., Yashin K.S. (2024). Peritumoral brain zone in astro-cytoma: Morphology, molecular aspects, and clinical manifestations (Review). Sovrem. Tekhnologii Med..

[20] Nejatie A., Yee S.S., Jeter A., Saragovi H.U. (2023). The cancer glycocode as a family of diagnostic biomarkers, exemplified by tu-mor-associated gangliosides. Front. Oncol..

[21] Mokbel K. (2024). GD2 in breast cancer: A potential biomarker and therapeutic target. Cancer Genom. Proteom..

[22] Balis F.M., Busch C.M., Desai A.V., Hibbitts E., Naranjo A., Bagatell R., Irwin M., Fox E. (2020). The ganglioside GD2 as a circulating tumor biomarker for neuroblastoma. Pediatr. Blood Cancer.

[23] Sanni A., Bennett A.I., Huang Y., Gidi I., Adeniyi M., Nwaiwu J., Kang M.H., Keyel M.E., Gao C., Reynolds C.P. (2024). An Optimized liquid chromatography-mass spectrometry method for ganglioside analysis in cell lines. Cells.

[24] Bartish M., Del Rincon S.V., Rudd C.E., Saragovi H.U. (2020). Aiming for the sweet spot: Glyco-immune checkpoints and gammadelta T cells in targeted immunotherapy. Front. Immunol..

[25] Sarbu M., Petrica L., Clemmer D.E., Vukelić Ž., Zamfir A.D. (2021). Gangliosides of human glioblastomamultiforme: A compre-hensive mapping and structural analysis by ion mobility tandem mass spectrometry. J. Am. Soc. Mass Spectrom..

[26] Zamfir A.D., Fabris D., Capitan F., Munteanu C., Vukelić Ž., Flangea C. (2013). Profiling and sequence analysis of gangliosides in human astrocytoma by high-resolution mass spectrometry. Anal. Bioanal. Chem..

[27] Sarbu M., Fabris D., Vukelić Ž., Clemmer D.E., Zamfir A.D. (2022). Ion mobility mass spectrometry reveals rare sialylatedglyco-sphingolipid structures in human cerebrospinal fluid. Molecules.

[28] Biricioiu M.R., Sarbu M., Ica R., Vukelić Ž., Kalanj-Bognar S., Zamfir A.D. (2024). Advances in mass spectrometry of gangliosides expressed in brain cancers. Int. J. Mol. Sci..

[29] Sarkar A., Banerjee S., Biswas K. (2023). Multi-dimensional role of gangliosides in modulating cancer hallmarks and their prospects in targeted cancer therapy. Front. Pharmacol..

[30] Nazha B., Inal C., Owonikoko T.K. (2020). Disialoganglioside GD2 expression in solid tumors and role as a target for cancer therapy. Front. Oncol..

[31] Cavdarli S., Groux-Degroote S., Delannoy P. (2019). Gangliosides: The double-edge sword of neuro-ectodermal derived tumors. Biomolecules.

[32] Groux-Degroote S., Delannoy P. (2021). Cancer-associated glycosphingolipids as tumor markers and targets for cancer immuno-therapy. Int. J. Mol. Sci..

[33] Shi M., Nan X.-R., Liu B.-Q. (2024). The multifaceted role of FUT8 in tumorigenesis: From pathways to potential clinical applications. Int. J. Mol. Sci..

[34] Sarbu M., Vukelić Ž., Clemmer D.E., Zamfir A.D. (2018). Ion mobility mass spectrometry provides novel insights into the expression and structure of gangliosides in the normal adult human hippocampus. Analyst.

[35] Liu J., Zheng X., Pang X., Li L., Wang J., Yang C., Du G. (2018). Ganglioside GD3 synthase (GD3S), a novel cancer drug target. Acta Pharm. Sin. B.

[36] Dobie C., Skropeta D. (2021). Insights into the role of sialylation in cancer progression and metastasis. Br. J. Cancer..

[37] Orozco-Moreno M., Visser E.A., Hodgson K., HipgraveEderveen A.L., Bastian K., Goode E.A., Öztürk Ö., Pijnenborg J.F.A., Eerden N., Moons S.J. (2023). Targeting aberrant sialylation and fucosylation in prostate cancer cells using potent metabolic inhibitors. Glycobiology.

[38] Svennerholm L., Fredman P. (1980). A procedure for the quantitative isolation of brain gangliosides. Biochim. Biophys. Acta.

[39] Vukelić Ž., Metelmann W., Muthing J., Kos M., Peter-Katalinić J. (2001). Anencephaly: Structural characterization of gangliosides in defined brain regions. Biol. Chem..

[40] Vukelić Ž., Kalanj-Bognar S., Froesch M., Bîndila L., Radić B., Allen M., Peter-Katalinić J., Zamfir A.D. (2007). Human gliosar-coma-associated ganglioside composition is complex and distinctive as evidenced by high-performance mass spectrometric determination and structural characterization. Glycobiology.

[41] Schiopu C., Flangea C., Capitan F., Serb A., Vukelić Ž., Kalanj-Bognar S., Sisu E., Przybylski M., Zamfir A.D. (2009). Determination of ganglioside composition and structure in human brain hemangioma by chip-based nanoelectrospray ionization tandem mass spectrometry. Anal. Bioanal. Chem..

[42] Schiopu C., Vukelić Ž., Capitan F., Kalanj-Bognar S., Sisu E., Zamfir A.D. (2012). Chip-nanoelectrosprayquadrupole time-of-flight tandem mass spectrometry of meningioma gangliosides: A preliminary study. Electrophoresis.

[43] Svennerholm L. (1980). Ganglioside designation. Adv. Exp. Med. Biol..

[44] Chester M.A. (1998). IUPAC-IUB joint commission on biochemical nomenclature (JCBN). Eur. J. Biochem..

[45] Domon B., Costello C.E. (1988). A systematic nomenclature for carbohydrate fragmentations in FAB-MS/MS spectra of glycoconju-gates. Glycoconj. J..

[46] Costello C.E., Juhasz P., Perreault H. (1994). New mass spectral approaches to ganglioside structure determinations. Prog. Brain Res..

[47] Ann Q., Adams J. (1992). Structure determination of ceramides and neutral glycosphingolipids by collisional activation of [M + Li] + ions. J. Am. Soc. Mass Spectrom..

