# Supplementary material for: Novel Application of Ion Mobility Mass Spectrometry Reveals Complex Ganglioside Landscape in Diffuse Astrocytoma Peritumoral Regions
Source: Int J Mol Sci. 2025 Aug 29;26(17):8433. doi: 10.3390/ijms26178433 (PMC12428295; doi:10.3390/ijms26178433)
Supplement: Supplementary file 1 [file ijms-26-08433-s001.zip › ijms-3749659-supplementary.pdf]

International Journal of
Molecular Sciences

MDPI

Article

Novel Application of Ion Mobility Mass Spectrometry Reveals Complex Ganglioside Landscape in Diffuse Astrocytoma Peritumoral Regions

Raluca Ica [1], Mirela Sarbu [1], Roxana Biricioiu [1,2], Dragana Fabris [3], Željka Vukelić [3] and Alina D. Zamfir [1,4,*]

1 National Institute for Research and Development in Electrochemistry and Condensed Matter, 300224 Timisoara, Romania; maria.biricioiu99@e-uvt.ro (R.B.)
2 Faculty of Physics, West University of Timisoara, 300223 Timisoara, Romania
3 Department of Chemistry and Biochemistry, School of Medicine, University of Zagreb, 10000 Zagreb, Croatia; zvukelic@mef.hr (Ž.V.)
4 Department of Technical and Natural Sciences, Aurel Vlaicu University of Arad, 310130 Arad, Romania
* Correspondence: alina.zamfir@uav.ro; Tel./Fax: +40-256-494413

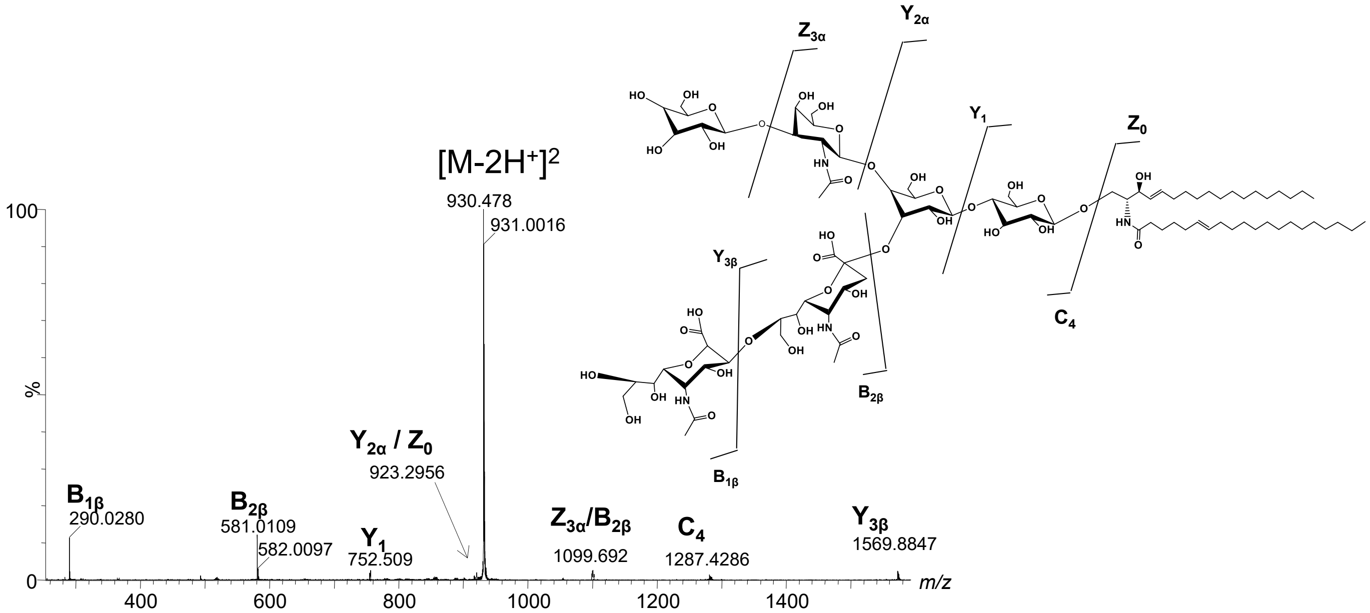

Figure S1. IMS MS/MS of the ion at *m/z* 930.478 corresponding to GD1(d18:1/20:1) ganglioside species isolated and fragmented from ganglioside mixture in peritumoral tissue of diffuse astrocytoma

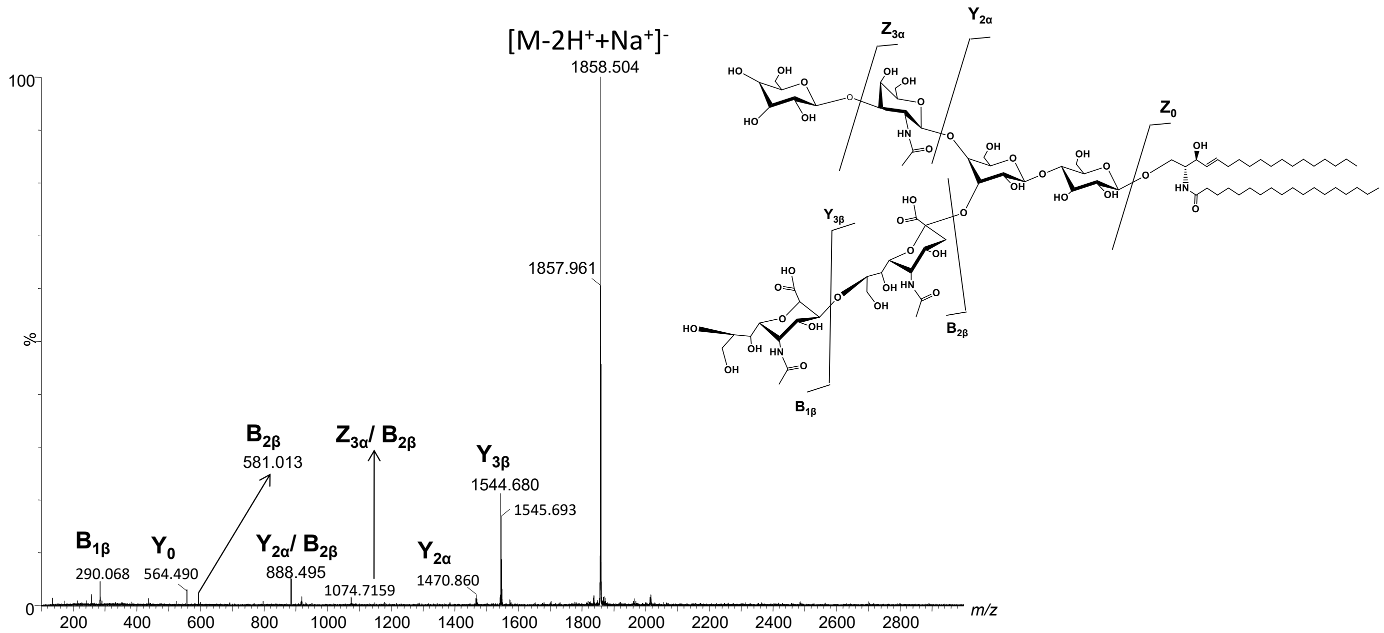

Figure S2. IMS MS/MS of the ion at *m*/*z* 1857.961 corresponding to sodiated GD1(d18:1/18:0) ganglioside species isolated and fragmented from ganglioside mixture in peritumoral tissue of diffuse astrocytoma

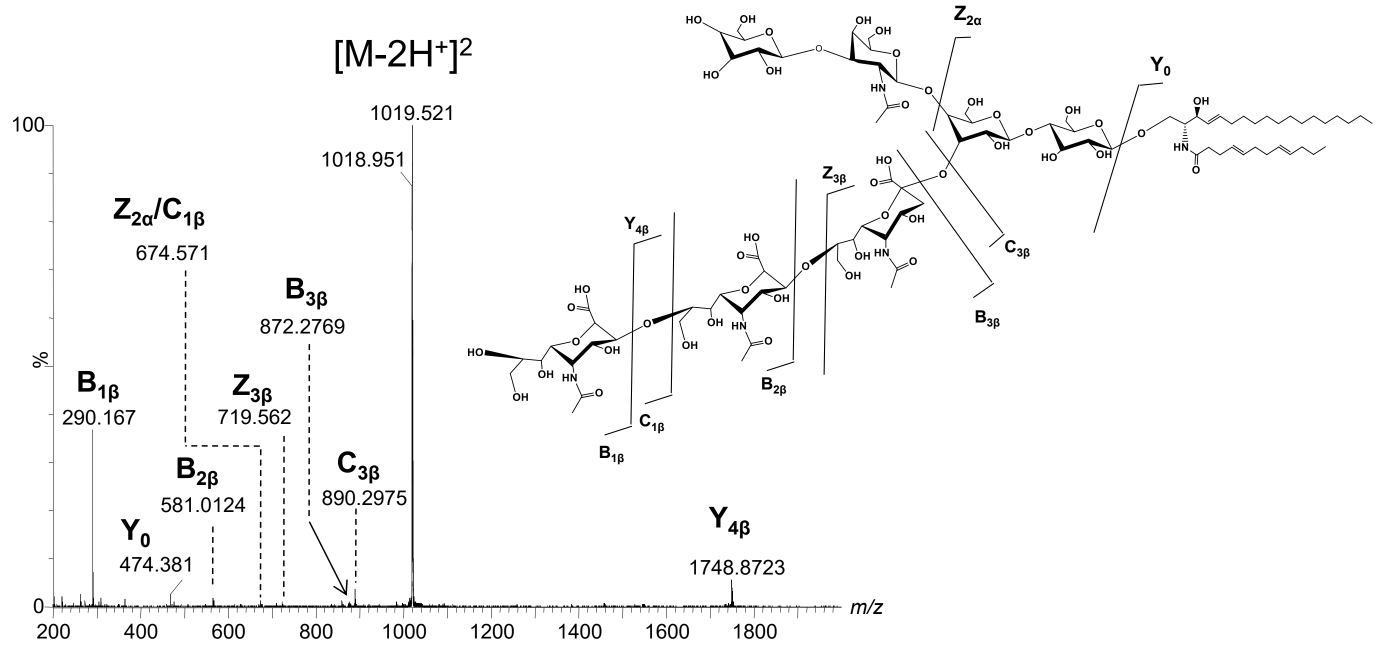

Figure S3. IMS MS/MS of the ion at *m*/*z* 1018.951 corresponding to GT1(d18:1/12:2) ganglioside species isolated and fragmented from ganglioside mixture in peritumoral tissue of diffuse astrocytoma

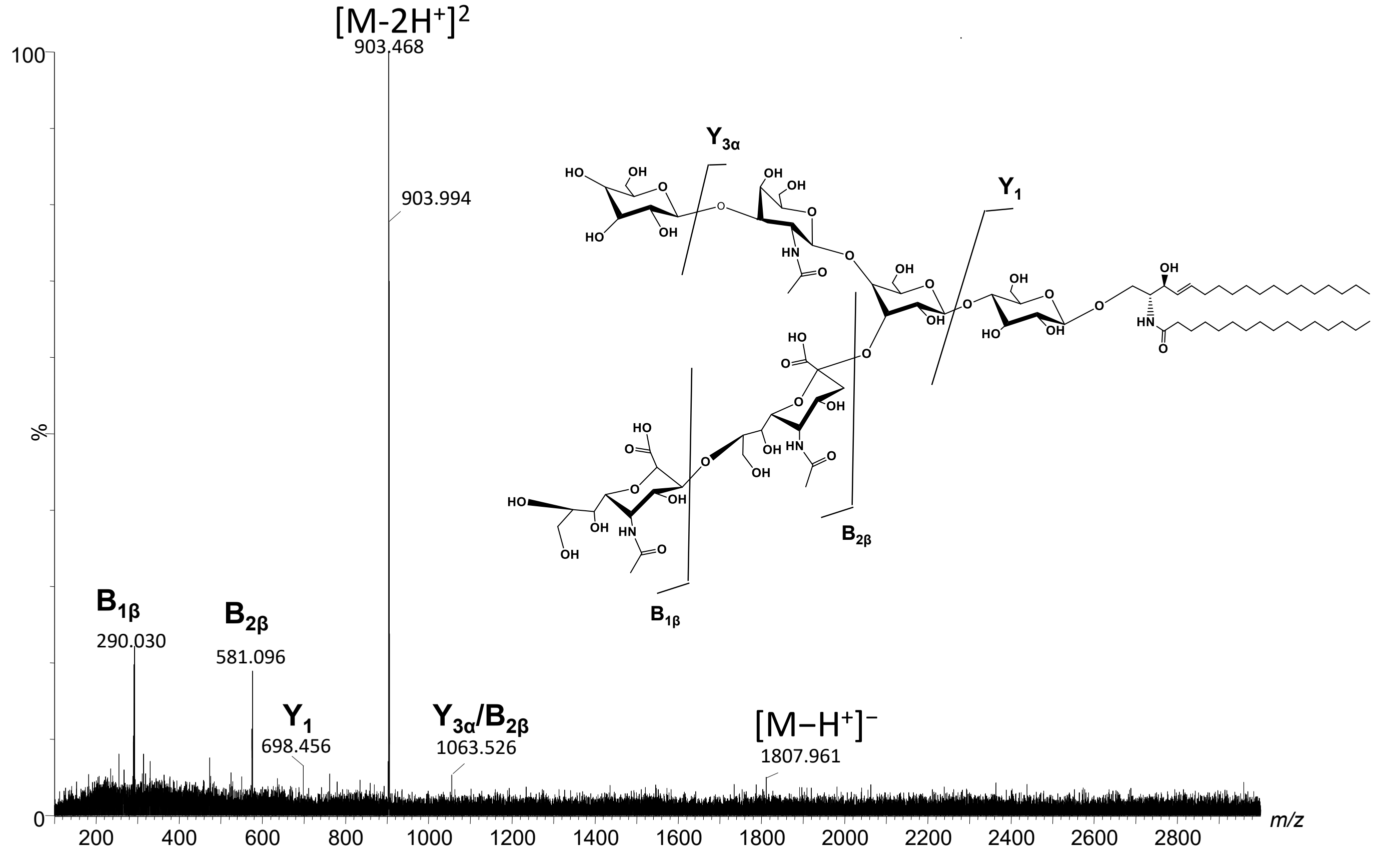

Figure S4. IMS MS/MS of the ion at *m/z* 903.468 corresponding to GD1(d18:1/16:0) ganglioside species isolated and fragmented from ganglioside mixture in peritumoral tissue of diffuse astrocytoma

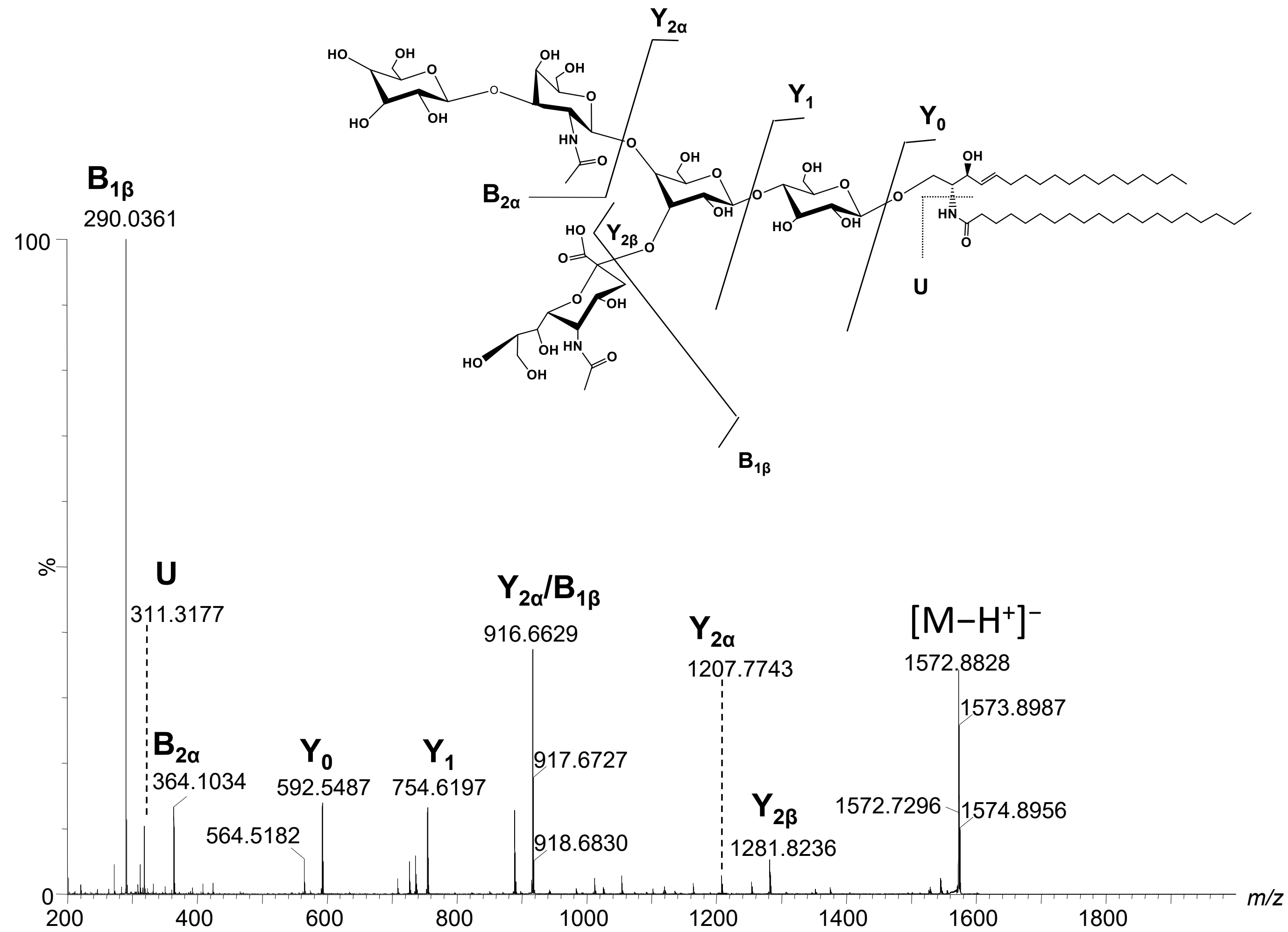

Figure S5. IMS MS/MS of the ion at *m/z* 1572.873 corresponding to GM1(d18:1/20:0) ganglioside species isolated and fragmented from ganglioside mixture in peritumoral tissue of diffuse astrocytoma
